# Transcatheter aortic valve implantation in patients with a small aortic annulus: performance of supra-, intra- and infra-annular transcatheter heart valves

**DOI:** 10.1007/s00392-021-01918-8

**Published:** 2021-08-13

**Authors:** Lisa Voigtländer, Won-Keun Kim, Victor Mauri, Alina Goßling, Matthias Renker, Atsushi Sugiura, Matthias Linder, Tobias Schmidt, Niklas Schofer, Dirk Westermann, Hermann Reichenspurner, Georg Nickenig, Stefan Blankenberg, Christian Hamm, Lenard Conradi, Matti Adam, Jan-Malte Sinning, Moritz Seiffert

**Affiliations:** 1 Department of Cardiology, University Heart and Vascular Center Hamburg, Hamburg, Germany; 2 Department of Cardiology, Kerckhoff Heart and Thorax Center, Bad Nauheim, Germany; 3 Department of Cardiology, Heart Center, University of Cologne, Cologne, Germany; 4 Department of Cardiology, University Heart Center, Bonn, Germany; 5 German Center for Cardiovascular Research (DZHK), Partner Site Hamburg/Lübeck/Kiel, Hamburg, Germany; 6 Department of Cardiovascular Surgery, University Heart and Vascular Center Hamburg, Hamburg, Germany

**Keywords:** Transcatheter aortic valve implantation, Aortic stenosis, Small aortic annulus

## Abstract

**Background:**

A small aortic annulus is associated with increased risk of prosthesis–patient mismatch (PPM) after transcatheter aortic valve implantation (TAVI). Whether specific transcatheter heart valve (THV) designs yield superior hemodynamic performance in these small anatomies remains unclear.

**Methods:**

Data from 8411 consecutive patients treated with TAVI from May 2012 to April 2019 at four German centers were retrospectively evaluated. A small aortic annulus was defined as multidetector computed tomography-derived annulus area < 400 mm^2^. TAVI was performed with a balloon-expanding intra-annular (Sapien-3, *n* = 288), self-expanding intra-annular (Portico, *n* = 110), self-expanding supra-annular (Evolut, *n* = 179 and Acurate-Neo, *n* = 428) and mechanically expanding infra-annular (Lotus, *n* = 64) THV according to local practice. PPM was defined as indexed effective orifice area ≤ 0.85cm^2^/m^2^.

**Results:**

A small annulus was found in 1069 (12.7%) patients. PPM was detected in 38.3% overall with a higher prevalence after implantation of a balloon-expanding intra-annular or mechanically expanding infra-annular THV compared to self-expanding intra- and supra-annular THV. Multivariable analysis linked self-expanding THV (Evolut: Odds ratio [OR] 0.341, Acurate-Neo: OR 0.436, Portico: OR 0.291), postdilatation (OR 0.648) and age (OR 0.968) to lower rates of PPM, while aortic valve calcification was associated with an increased risk (OR 1.001). Paravalvular regurgitation > mild was more frequent after TAVI with self-expanding THV (*p* = 0.04).

**Conclusion:**

In this large contemporary multicenter patient population, a substantial number of patients with a small aortic anatomy were left with PPM after TAVI. Self-expanding supra- and intra-annular THV demonstrated superior hemodynamics in these patients at risk, however at the cost of higher rates of residual paravalvular regurgitation.

**Graphic abstract:**

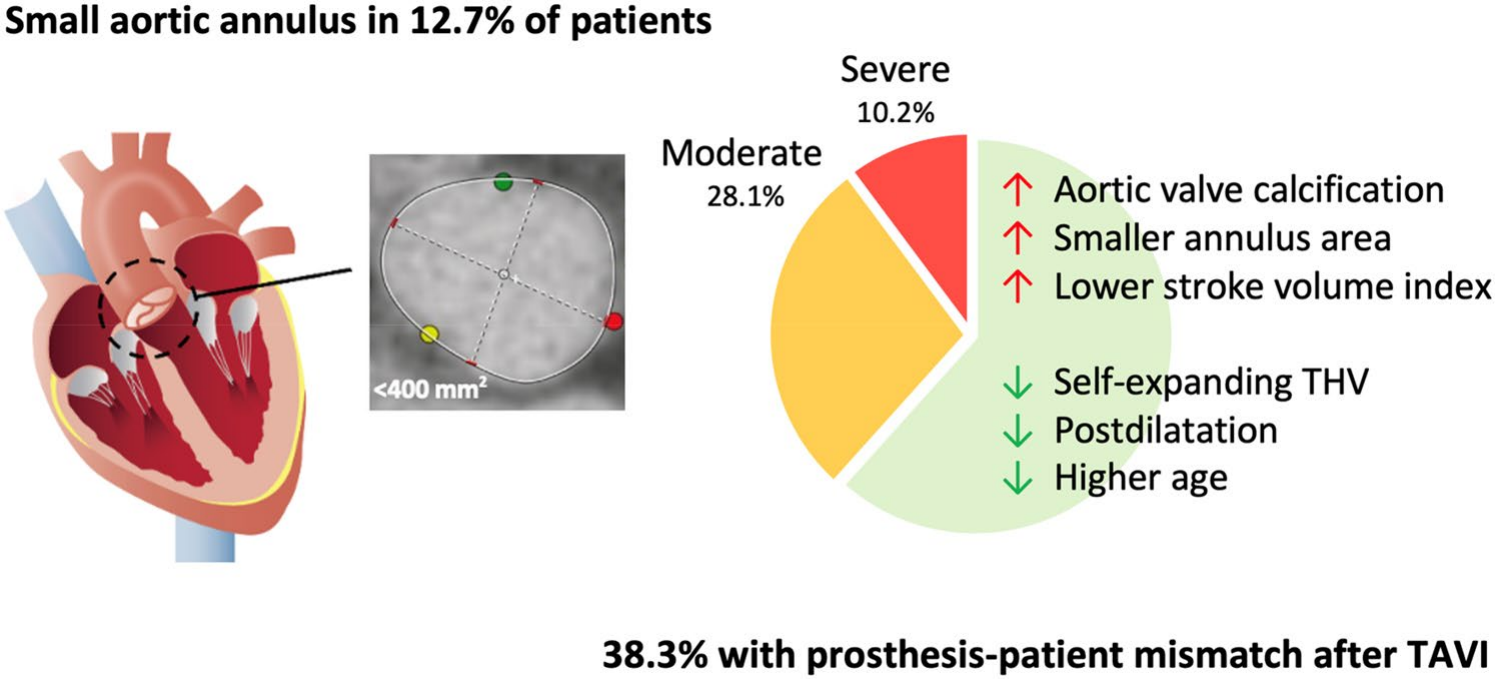

**Supplementary Information:**

The online version contains supplementary material available at 10.1007/s00392-021-01918-8.

## Introduction

Patient–prosthesis mismatch (PPM) after aortic valve replacement is associated with increased long-term mortality and morbidity [1, 2]. Several strategies were developed to minimize the risk of PPM after surgical aortic valve replacement (SAVR), i.e. aortic root enlargement or implantation of sutureless bioprostheses [3, 4]. Although lower rates of PPM were reported after transcatheter aortic valve implantation (TAVI) [5, 6], the prevalence of moderate and severe PPM was described in as many as 35% of patients [7]. Among other risk factors (i.e. obesity and impaired left ventricular function), a small native aortic annulus was associated with increased rates of PPM [7]. Recent analyses favored supra-annular transcatheter heart valves (THV) for the treatment of patients with severe aortic stenosis and a small aortic annulus (SAA) [8–10]. However, several additional THV designs have become commercially available in the meantime and optimal valve selection for patients with SAA remains unclear.

Aims of our study were (i) to determine the frequency of small aortic anatomies in a consecutive patient population scheduled for TAVI, (ii) to assess the prevalence of prosthesis patient mismatch in these patients, (iii) to detect risk factors for prosthesis patient mismatch and (iv) to compare hemodynamic performance of supra-, intra- and infra-annular THV designs to gain further insights into improved treatment options for these patients at risk.

## Methods

### Patient population

This retrospective analysis included transfemoral TAVI procedures performed between 2012 and 2019 with balloon-expanding (BE: Edwards Lifesciences Sapien-3), self-expanding (SE: Medtronic Evolut/Evolut-R/Pro, Boston Scientific Acurate-Neo, Abbott Vascular Portico) and mechanically expanding (ME: Boston Scientific Lotus) devices at four high-volume centers in Germany: University Heart Center Hamburg, Kerckhoff Heart and Thorax Center Bad Nauheim, University Heart Center Bonn and University hospital Cologne. In accordance with previous studies [8, 9], patients with a SAA (defined as annulus area < 400 mm^2^, measured by multidetector computed tomography (MDCT)) were included. Patients with valve-in-valve procedures, treatment of predominant aortic regurgitation and procedures via non-transfemoral access routes were excluded. Patients were divided into five groups according to the implanted THV (Sapien-3, Evolut, Acurate-Neo, Portico and Lotus).

### Transcatheter aortic valve implantation

All cases were reviewed by the local heart team. After written informed consent was obtained, TAVI and selection of THV were performed according to local practice and expertise. Transthoracic echocardiography with assessment of mean valvular gradients, effective orifice areas (EOA) according to the continuity equation and aortic regurgitation were performed before TAVI and at discharge. Baseline contrast-enhanced MDCT imaging was analyzed with the 3mensio software (3mensio Structural Heart, 3mensio Medical Imaging, Maastricht, The Netherlands) with a focus on annulus geometry and landing zone calcification, which contained the aortic valve complex, defined as the region between basal plane and coronary ostium, and left ventricular outflow tract (LVOT), defined as the region from 5 mm inferior to basal plane [11]. The implantation depth was assessed at the left coronary cusp and non-coronary cusp in the final angiography in a perpendicular annular plane. Implantation depth was defined as the distance between native aortic annulus and the end of the stent frame (inflow) in the LVOT.

### Data acquisition and endpoint definitions

Data acquisition was performed by the local study team, and endpoint assessment was performed by an experienced interventional cardiologist at the local study site. The primary endpoint was the occurrence of moderate or severe PPM after TAVI. PPM was defined according the Valve Academic Research Consortium criteria (VARC-2) as moderate (indexed EOA (iEOA) ≤ 0.85 cm2) and severe (iEOA ≤ 0.65 cm2) with correction in obese patients (body mass index ≧30 kg/m2; moderate PPM: iEOA < 0.7 cm2/m2; severe PPM: iEOA < 0.6 cm^2^/m^2^) [12]. Secondary endpoints and echocardiographic outcomes were defined according to VARC-2 criteria [12]. All patients provided informed consent to the procedure and data acquisition. Ethics committee approval was obtained according to local requirements. The study was performed in accordance with the 1964 Declaration of Helsinki and its later amendments.

### Statistical analysis

Continuous variables were shown as median (interquartile range [IQR]), whereas binary variables were shown as absolute numbers and percentages. For between-group comparisons, the Mann–Whitney test was used for continuous variables and the *c*^2^ test for binary variables. Uni- and multivariable logistic regression was performed twice: (i) for moderate or severe PPM and (ii) for severe PPM. First, we selected variables with a *p* value < 0.2 from Tables 1 and 2. Variables used to derive PPM (e.g. BSA or iEOA) or with significant missings were excluded, and certain variables were added due to clinical relevance. This led to the following set of variables: (i) age, male sex, BMI, prior malignancy, stroke volume index (SVI), annulus area, sizing in the categories “Within normal range”, “Oversizing” and “Undersizing”, LVOT calcification, AVC calcification, THV groups, mean transaortic gradient and postdilatation to predict moderate or severe PPM and (ii) age, male sex, BMI, NYHA class, SVI, annulus area, sizing in the categories “Within normal range”, “Oversizing” and “Undersizing”, LVOT calcification, AVC calcification, THV groups (Sapien-3 as reference, Evolut, Acurate-Neo, Portico and Lotus), mean transaortic gradient and postdilatation. These variables were analyzed by univariate logistic regression analysis and were included in multivariable logistic regression analysis if *p* values were < 0.05. The regression was additionally adjusted for the different centers.

**Table 1:** Baseline characteristics

	All (*n* = 1069)	Sapien 3 (*n* = 288)	Evolut (*n* = 179)	Acurate Neo (*n* = 428)	Portico THV(*n* = 110)	Lotus (*n* = 64)	*p* value
Clinical data							
Age (years)	82.8 (79.4–86.1)	82.8 (78.8–85.9)	83.0 (79.8–6.8)	82.4 (79.4–85.7)	83.3 (80.2–85.8)	83.0 (80.0–86.7)	0.52
Female gender (%)	994 (93)	264 (91.7)	168 (93.9)	398 (93.0)	106 (96.4)	58 (90.6)	0.49
BSA (m^2^)	1.7 (1.6–1.8)	1.7 (1.6–1.8)	1.7 (1.6–1.8)	1.7 (1.6–1.8)	1.7 (1.6–1.8)	1.7 (1.6–1.8)	0.22
CAD (%)	574 (53.8)	164 (56.9)	97 (54.2)	230 (54.0)	56 (50.9)	27 (42.9)	< 0.001
COPD (%)	184 (17.2)	54 (18.8)	34 (19.0)	78 (18.2)	12 (10.9)	6 (9.4)	< 0.001
Diabetes (%)	266 (24.9)	69 (24.0)	38 (21.2)	110 (25.7)	37 (33.6)	12 (18.8)	< 0.001
Stroke (%)	133 (12.4)	43 (14.9)	15 (8.4)	52 (12.1)	14 (12.7)	9 (14.1)	< 0.001
STS PROM (%)	4.5 (3.1–6.5)	4.5 (3.2–6.6)	4.7 (3.2–6.5)	4.4 (3.2–6.5)	4.9 (3.1–6.6)	4.0 (3.0–5.2)	0.33
Echocardiographic and CT data							
LVEF < 30%	708 (66.4)	180 (62.7)	133 (74.7)	294 (68.9)	74 (67.3)	27 (42.2)	< 0.001
Stroke volume index (ml/m^2^)	48.0 (37.8–66.0)	46.5 (36.3–61.0)	41.9 (32.6–47.3)	56.0 (41.0–69.0)	57.3 (42.3–5.0)	41.7 (34.6–52.5)	< 0.001
iEOA (cm^2^/m^2^)	0.4 (0.3–0.5)	0.4 (0.3–0.5)	0.4 (0.3–0.5)	0.4 (0.3–0.5)	0.4 (0.3–0.4)	0.4 (0.3–0.5)	0.34
Mean gradient (mmHg)	42.0 (32.0–53.0)	42.0 (31.8–53.0)	40.0 (32.0–48.0)	42.0 (32.0–54.0)	44.0 (32.0–56.0)	44.0 (33.1–57.0)	0.12
CT Annulus Area (mm^2^)	368.2 (342.0,–383.1)	376.1 (351.9–386.2)	359.8 (320.9–379.8)	368.0 (345.4–382.9)	361.5 (331.9–380.8)	370.0 (353.9–388.3)	< 0.001
CT Annulus Perimeter (mm)	69.4 (67.0–71.0)	70.0 (68.0–71.2)	68.4 (64.9–70.1)	69.5 (67.2–71.0)	68.8 (66.6–70.7)	70.0 (68.5–71.3)	< 0.001
AVC calcification (mm^3^)	399.0 (227.2–623.7)	454.5 (272.9–716.8)	341.0 (190.8–579.7)	355.8 (177.3–557.5)	472.7 (271.3–681.5)	573.3 (297.5–877.7)	< 0.001
LVOT calcification (mm^3^)	5.0 (0–35.1)	10.0 (0–44.2)	12.1 (0.1–76.7)	0.4 (0–16.3)	5.3 (0–32.6)	22.1 (0.1–82.4)	< 0.001

*AVC* aortic valve complex, *BSA* body surface area, *CAD* coronary artery disease, *COPD* chronic obstructive lung disease, *CT* computed tomography, *iEOA* indexed effective orifice area, *LVEF* left ventricular ejection fraction, *LVOT* left ventricular outflow tract

**Table 2:** Procedural aspects

	All (*n* = 1069)	Sapien 3 (*n* = 288)	Evolut (*n* = 179)	Acurate Neo (*n* = 428)	Portico (*n* = 110)	Lotus (*n* = 64)	*p* value
THV design		BE intra-annular	SE supra-annular	SE supra-annular	SE intra-annular	ME infra-annular	
Median THV size (mm)	23.0 (23.0–25.0)	23.0 (23.0–23.0)	26.0 (26.0–26.0)	S: 342 (79.9%) M: 86 (20.1%) L:0	25.0 (23.0–25.0)	23.0 (23.0–23.0)	< 0.001
Sizing (according to IFU)							
Undersizing (%)	26 (2.5)	0 (0)	21 (12.4)	0 (0)	5 (4.7)	0 (0)	< 0.001
Within normal range (%)	750 (72.3)	225 (83.6)	143 (84.1)	237 (55.5)	97 (90.7)	48 (75.0)	< 0.001
Oversizing (%)	261 (25.2)	44 (16.4)	6 (3.5)	190 (44.5)	5 (4.7)	16 (25.0)	< 0.001
Relative oversizing (%)	21.7 (10.0–33.4)	8.8 (5.5–18.4)	44.7 (39.0–57.0)	18.7 (11.3–25.9)	30.3 (25.9–36.4)	15.0 (10.4–24.5)	< 0.001
Local anesthesia /conscious sedation (%)	829 (78.7)	189 (68.0)	153 (86.4)	355 (83.1)	77 (71.3)	55 (85.9)	< 0.001
Procedure time (min)	52.0 (35.0–75.0)	52.5 (32.0–80.1)	61.0 (47.9–86.1)	41.5 (31.0–65.6)	50.0 (37.9–70.0)	83.0 (57.2–109.6)	< 0.001
Contrast media (ml)	115.0 (80.0–164.1)	102.5 (70.0–150.6)	140.5 (117.8–180.0)	95.0 (70.0–140.0)	136.0 (100.0–200.2)	143.0 (110.8–212.2)	< 0.001
Predilatation (%)	570 (53.5)	113 (39.2)	45 (25.1)	294 (69.2)	86 (78.2)	32 (50.8)	< 0.001
Postdilatation (%)	266 (25.0)	37 (12.8)	46 (25.7)	134 (31.5)	46 (41.8)	3 (4.8)	< 0.001

*BE* balloon-expandable, *IFU* instructions for use, *ME* mechanically expanding, *SE* self-expanding, *THV* transcatheter heart valve

## Results

### Patient characteristics

Between May 2012 and April 2019, 8411 consecutive patients underwent transfemoral TAVI for severe aortic stenosis; 1069 patients (12.7%) had a SAA and were included in this study. TAVI was performed with Sapien-3 THV in 288 (27%), with Evolut THV in 179 (17%), with Acurate-Neo THV in 428 (40%), with Portico THV in 110 (10%) and with Lotus THV in 64 (6%) patients, respectively.

The majority of the patients were female (93%) with a median age of 82.8 [79.4, 86.1] years. Despite a similar risk profile according to the STS PROM score, comorbidities differed significantly among the groups (Table 1). Preprocedural iEOA and mean aortic gradients were comparable, but patients treated with SE THV had significantly smaller annulus areas and perimeters compared to patients treated with BE or ME THV. Calcium distribution also differed among patients: calcification of the aortic valve complex was more severe in the Lotus, Portico and Sapien-3 groups, while LVOT-calcification was particularly severe in patients treated with Lotus THV (Table 1).

### Procedural aspects

Procedures were predominantly performed in local anesthesia or conscious sedation (79.6%, Table 2). Procedural duration was longer with application of more contrast media in patients treated with Evolut and Lotus THV. Relative oversizing was more frequent in patients treated with SE (Evolut: 44.7 [39.0–57.0] %, Portico: 30.3 [25.9–36.4] %, Acurate-Neo: 18.7 [11.3–25.9] %) compared to ME (15.0 [10.4–24.5] %) and BE THV (8.8 [5.5–18.4] %, *p* < 0.001). Oversizing exceeding the instructions for use (IFU) was more common in patients treated with Acurate-Neo (44.5%), Lotus THV (25.0%) and Sapien-3 (16.4%) compared to Portico (4.7%) and Evolut THV (3.5%, *p* < 0.001). Predilatation was performed in 53.5% of procedures overall, while postdilatation was performed particularly after SE vs. BE or ME THV implantation (31.5% vs. 11.4%, *p* < 0.001, Table 2).

### Echocardiographic and clinical outcome

Echocardiographic and clinical outcomes are reported in Table 3. iEOA was larger and mean valvular gradients were lower after TAVI with SE compared to patients treated with BE and ME THV. More than mild PVL was observed in 7.0% (Acurate-Neo), 5.5% (Portico), 3.9% (Evolut), 2.4% (Sapien-3) and 1.6% (Lotus), respectively (*p* = 0.04).

**Table 3:** Clinical outcomes

	All (*n* = 1069)	Sapien 3 (*n* = 288)	Evolut (*n* = 179)	Acurate Neo (*n* = 428)	Portico (*n* = 110)	Lotus (*n* = 64)	*p* value
Echocardiographic outcome							
Moderate PPM	243 (28.1)	93 (40.7)	28 (21.9)	95 (25.2)	20 (21.1)	7 (16.7)	< 0.001
Severe PPM	91 (10.2)	38 (15.8)	12 (8.8)	26 (7.4)	4 (4.4)	11 (22.2)	< 0.001
iEOA (cm^2^/m^2^)	0.9 (0.7–1.1)	0.8 (0.7–1.0)	1.0 (0.8–1.1)	0.9 (0.8–1.1)	0.9 (0.8–1.1)	0.9 (0.6–1.1)	< 0.001
Mean gradient (mmHg)	10.0 (6.8–13.0)	12.2 (9.0–16.0)	7.0 (4.9–10.2)	9.0 (6.0–12.0)	9.0 (7.0–11.0)	12.9 (9.8–16.0)	< 0.001
PVL > mild (%)	51 (4.8)	7 (2.4)	7 (3.9)	30 (7.0)	6 (5.5)	1 (1.6)	0.04
VARC-2 clinical outcome							
Major vascular complications (%)	78 (7.3)	14 (4.9)	4 (2.2)	44 (10.3)	10 (9.1)	6 (9.4)	0.003
Acute kidney injury (%)^a^	84 (8.3)	20 (8.3)	18 (10.3)	32 (7.6)	11 (10.0)	3 (4.8)	0.63
Major Bleeding (%)	67 (6.3)	17 (5.9)	5 (2.8)	30 (7.0)	9 (8.2)	6 (9.4)	0.21
Permanent pacemaker implantation (%)	134 (13.8)	43 (16.5)	16 (9.8)	41 (10.6)	18 (17.8)	16 (27.6)	0.001
Disabling Stroke (%)	8 (0.7)	1 (0.3)	2 (1.1)	3 (0.7)	1 (0.9)	1 (1.6)	0.81
Reintervention (%)^b^	3 (0.3)	1 (0.4)	0 (0)	2 (0.5)	0 (0)	0 (0)	0.78
30-day mortality (%)	33 (3.2)	9 (3.2)	7 (4.0)	9 (2.2)	5 (4.6)	3 (4.8)	0.54
12-month mortality (%)	104 (9.9)	31 (11.0)	16 (9.1)	38 (9.1)	11 (10.1)	8 (12.9)	0.84

*iEOA* indexed effective orifice area, *PVL* paravalvular leakage, *PPM* prosthesis-patient mismatch

^a^Acute kidney injury `Kidney Disease—Improving Global Outcomes` stadium I–III

^b^Reasons for reintervention: relevant aortic regurgitation after postdilatation

Peri- and postprocedural complications were similar, except for lower rates of major vascular complications in patients treated with Sapien-3 (4.9%) and Evolut (2.2%) compared to Acurate-Neo (10.3%), Portico (9.1%) and Lotus THV (9.4%, *p* = 0.003) and significant differences in permanent pacemaker implantations after TAVI. Patients treated with Lotus THV had the highest rates of permanent pacemaker implantations (27.6%) and patients treated with supra-annular self-expanding THV (Evolut: 9.8%, Acurate-Neo: 10.6%) had lower permanent pacemaker rates compared to patients treated with intra-annular self- or balloon-expanding THV (Sapien-3: 16.5%, Portico: 17.8%, *p* = 0.001). 30-day and 12-month mortality rates were 3.2% and 9.9%, respectively, without differences between the THV groups.

### Prosthesis–patient mismatch

**Fig. 1:**
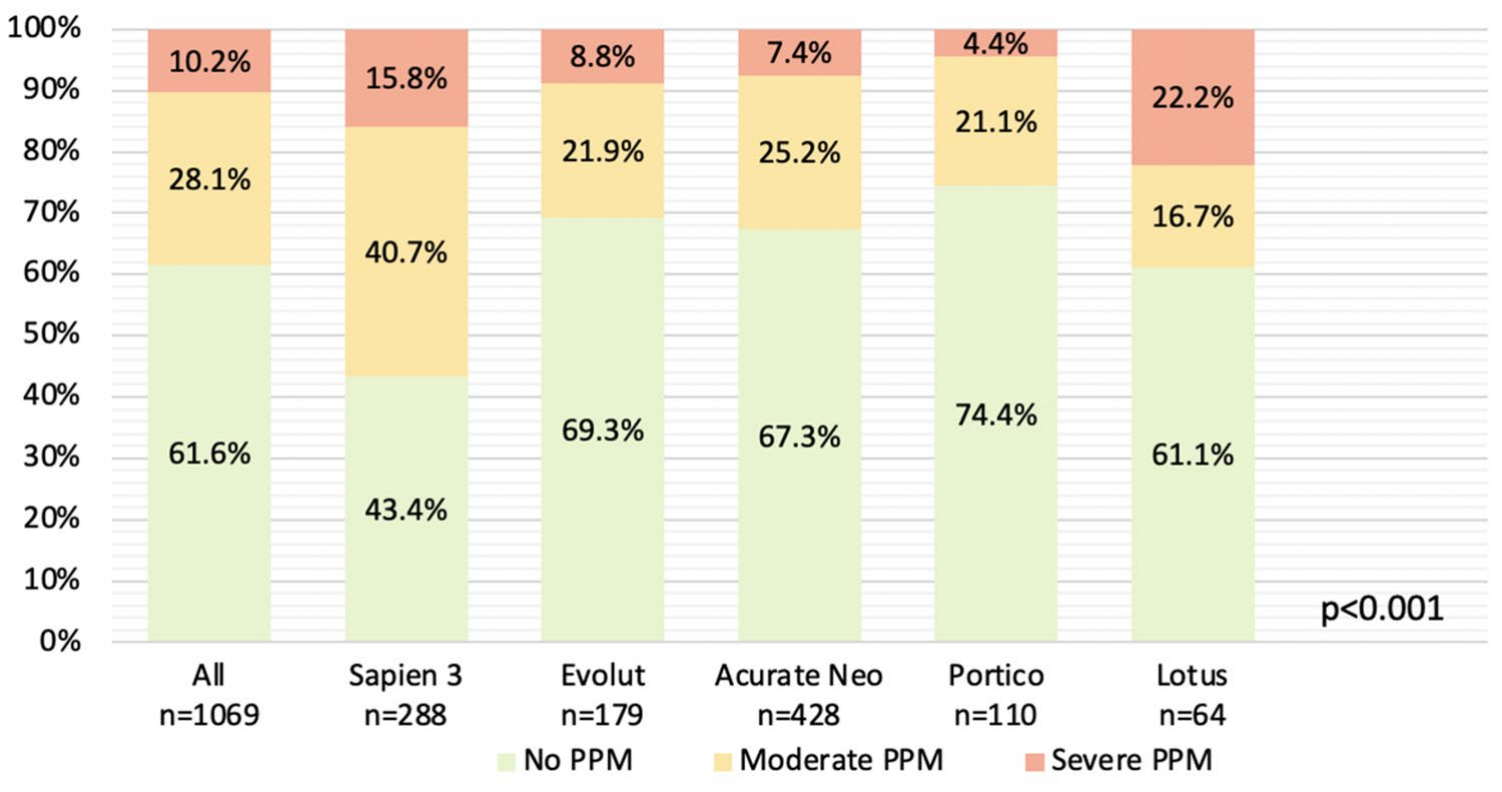
Prevalence of moderate and severe PPM with different devices. *p* < 0.001 for moderate and severe PPM. *PPM* prosthesis–patient mismatch

The prevalence of PPM was 38.3% overall (moderate PPM: 28.1% and severe PPM: 10.2%) (Fig. 1). Patients treated with Sapien-3 and Lotus THV had higher rates of PPM (Sapien-3: 56.5%, Lotus: 38.9%) compared to patients treated with Evolut (30.7%), Acurate-Neo (32.6%) and Portico THV (25.5%, *p* < 0.001). Severe PPM occurred most frequently after Lotus (22.2%) and Sapien-3 (15.8%) implantation. In patients treated with Evolut, Acurate-Neo and Portico THV, rates of severe PPM were 8.8%, 7.4% and 4.4%.

Patients with severe PPM had a larger body surface area, smaller SVI and iEOAs, higher transvalvular gradients and more severe aortic valve calcification at baseline (*p* < 0.001, Table S1). Postdilatation was less common in patients with PPM (severe PPM: 18.7%, moderate PPM: 21.1%, no PPM: 29.3%, *p* = 0.014, Table S2). Clinical outcomes were comparable in patients with and without PPM; however, a trend toward higher rates of acute kidney injury was observed in patients with severe PPM (severe PPM: 13.8%, moderate PPM: 8.5%, no PPM: 6.8%, *p* = 0.080, Table S3). Mortality at 30 days and 1 year did not differ significantly between patients with and without PPM (Table S3).

**Fig. 2:**
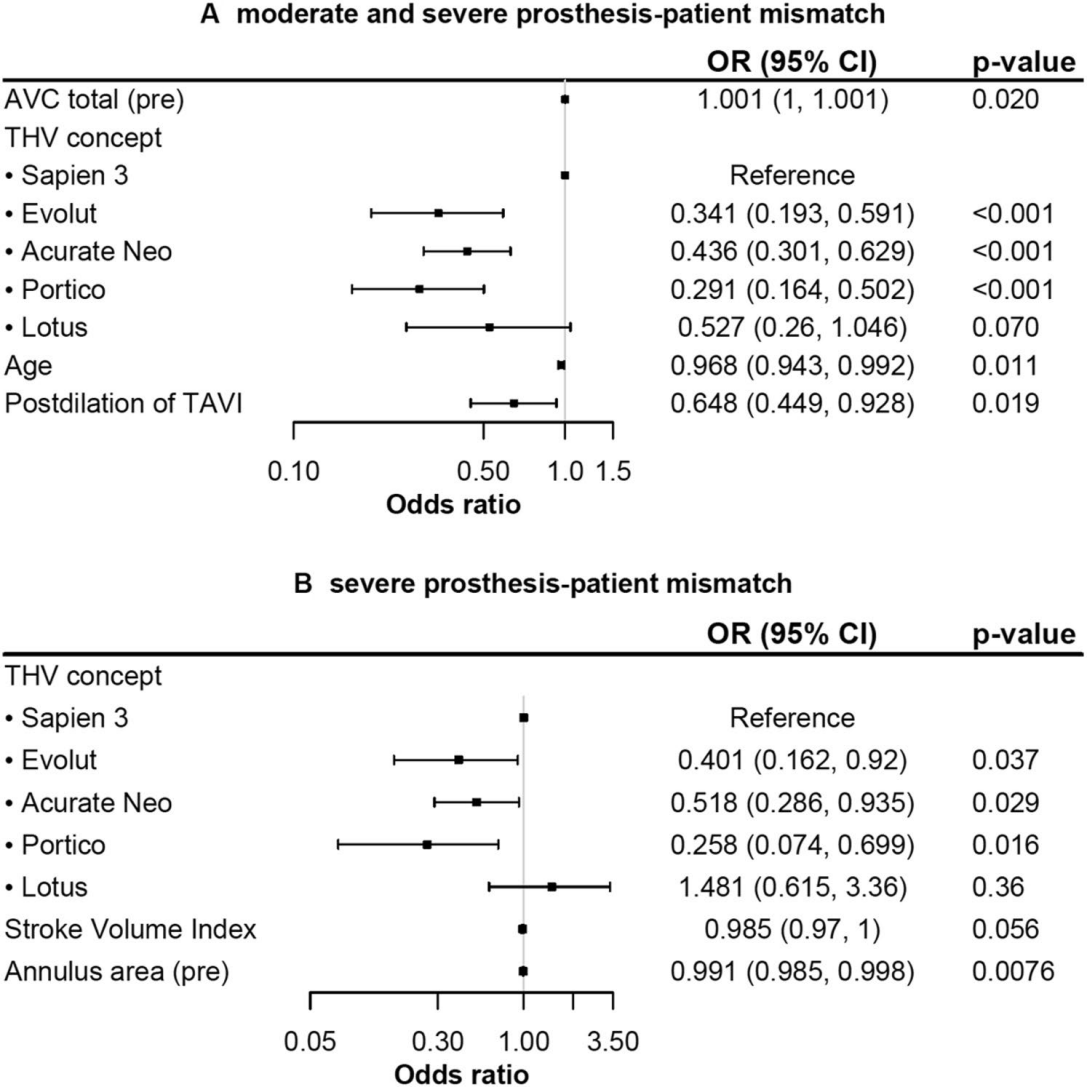
Predictors for moderate and severe (**A**) and severe (**B**) PPM. Multivariable analysis for predictors of (**A**) moderate/severe and (**B**) severe prosthesis–patient mismatch. *AVC* aortic valve complex, *OR* odds ratio, *THV* transcatheter heart valve

Multivariable analysis identified higher age (OR 0.968), SE THV (Evolut: OR 0.341, Acurate-Neo: OR 0.436, Portico: OR 0.291) and postdilatation (OR 0.648) to be associated with less moderate or severe PPM. Aortic valve complex calcification increased the risk of PPM (OR 1.001, Fig. 2). Severe PPM was observed more often in patients with a smaller annulus area (OR 0.991) and a trend to a lower SVI (OR 0.985). SE THV was associated with less severe PPM (Fig. 2).

**Fig. 3:**
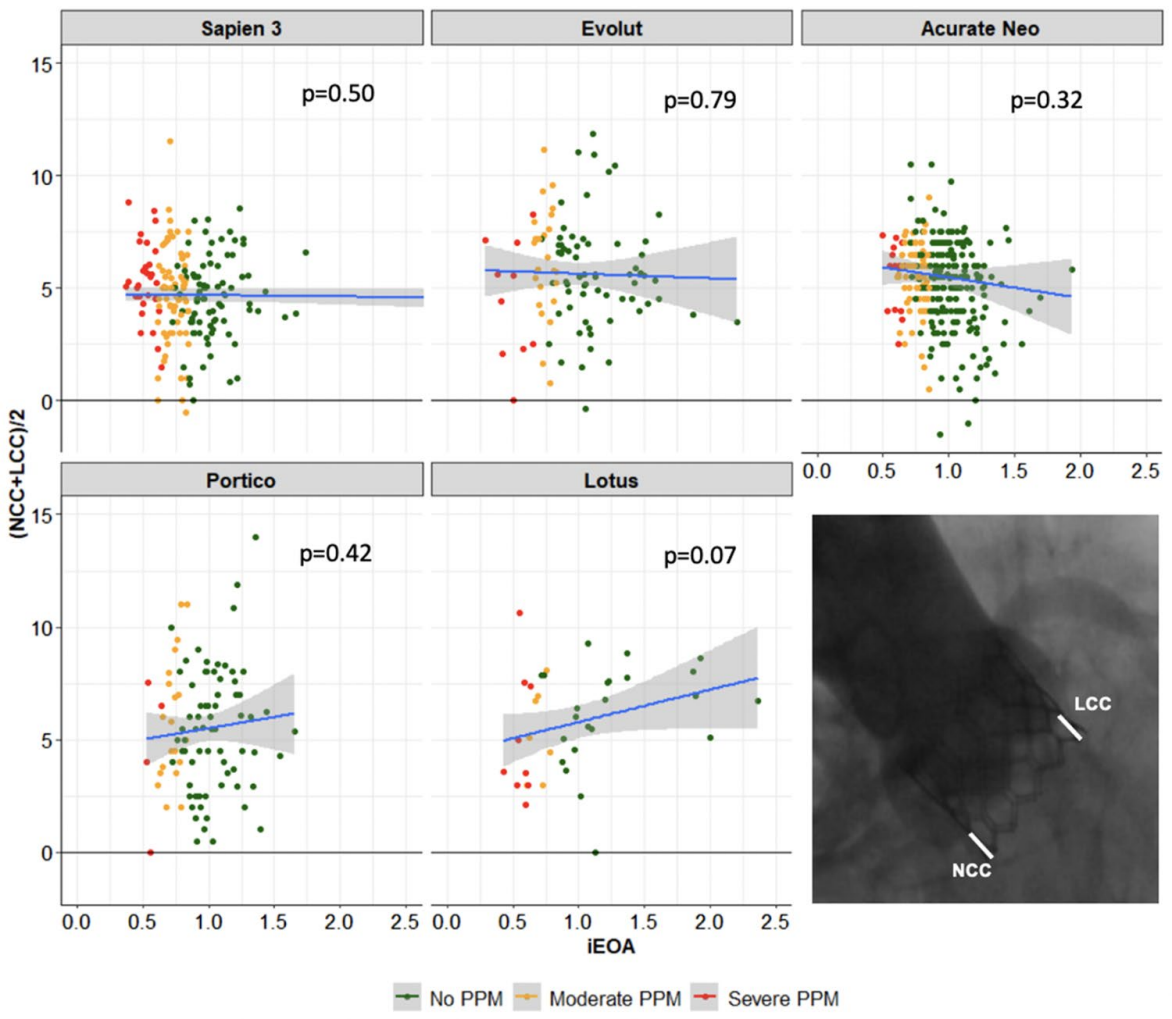
Association of implantation depth and iEOA. *iEOA* indexed effective orifice area, *LCC* left coronary cusp, *NCC* non-coronary cusp

We did not find any association of implantation depth and iEOA in any of the THV platforms assessed (Fig. 3). Evaluation of implant depth by tertiles did not render any association with the occurrence of PPM or PVL (Table S4).

## Discussion

This retrospective multicenter study examined the hemodynamic results and clinical outcomes of five different THV platforms in 1,069 patients with a SAA. The main findings were: (i) 12.7% of patients treated with TAVI had a SAA (< 400 mm^2^), the vast majority being female. (ii) Prosthesis–patient mismatch occurred in 38.3% of these patients with a significantly lower prevalence after implantation of a SE supra- or intra-annular THV. (iii) Calcification of the aortic valve complex, a smaller aortic annulus area and lower SVI were additionally associated with PPM, and less PPM was observed in older patients and if postdilatation of the THV was performed.

The presence of a SAA in patients with severe aortic stenosis poses a challenge on further management and is particularly common in women [13]. A prevalence of up to 40% of patients undergoing TAVI was reported for the Asian OCEAN-TAVI-registry [10]. However, these numbers may vary largely with variable definitions and variations in patient populations [14], potentially explaining the difference to the 12.7% prevalence identified in our multicenter analysis.

The risk of PPM is considerable in these patients, particularly after SAVR [5]. The negative impact of PPM on outcome has been reported in previous studies [2, 15]. A meta-analysis comprising 27,186 patients after SAVR found moderate and severe PPM to significantly increase all-cause mortality over a long-term follow-up of more than five years (moderate PPM: HR 1.19, 95% CI 1.07–1.33; severe PPM: HR = 1.84, 95% CI 1.38–2.45) [15]. Potentially due to smaller patient numbers and shorter follow-up, we were not able to replicate this association in our study population. Longer observation may validate a potential adverse impact on long-term survival.

Due to improved hemodynamics after TAVI compared to SAVR in patients with small aortic anatomies [5], TAVI has increasingly been promoted for the treatment of patients at risk to avoid PPM. Except for the presence of a SAA, however, risk factors for PPM after TAVI in these patients remain largely unclear. In addition to THV design, we found calcification of the aortic valve complex and younger age to be associated with moderate or severe PPM while patients with postdilatation after TAVI were at lower risk.

Severe PPM was linked to a lower SVI, a smaller annulus area and—again—to the THV design. Reduced left ventricular ejection fraction is a known risk factor for PPM [16] and supports our findings on low SVI. This is of clinical relevance as patients with PPM and reduced left ventricular function have particularly impaired outcomes [17]. Younger age as a risk factor for PPM has been described before [5]. However, the reason for this still remains unclear.

Our main finding was that SE THV design was independently associated with less PPM which was robust among several multivariate analyses. The hemodynamic advantage of a SE supra-annular THV (Acurate-Neo) over BE intra-annular THV (Sapien-3) in patients with a SAA was reported before [8]. The TAVI-SMALL registry recently demonstrated similar PPM rates with three SE THV platforms (Evolut/Pro, Acurate and Portico) [9] which were in line with the prevalence observed in our analysis. Beyond that, we compared the performance of 5 current THV platforms to further investigate hemodynamic results in patients with a SAA. Moderate and particularly severe PPM was less frequent after implantation of SE (supra- and intra-annular) THV compared to BE (intra-annular) or ME (infra-annular) devices. Interestingly, we did not observe any differences between SE supra- or intra-annular THV designs, contradicting the idea that supra-annular seating alone yields improved hemodynamics. Even though Sapien-3 and Portico THV both feature intra-annular designs, the latter revealed lower rates of PPM. Whether additional materials (e.g. sealing skirts) impact this aspect remains to be determined. Interestingly, implantation depth alone did not correlate with iEOA or the occurrence of PPM for any of the evaluated THV. Hence, THV design rather than implantation technique may be important to avoid PPM in patients at risk.

Due to its adverse impact on outcomes, further refinement of devices and implantation techniques aims to minimize residual regurgitation [18]. Aortic valve calcification is a well-known risk factor for PVL [11] and our data add a link to PPM, making patients with severely calcified aortic valves and SAA a particularly vulnerable patient population. Relevant PVL was more frequent after implantation of a SE THV—an aspect that must be weighted against the benefits achieved with lower rates of PPM. Several device iterations have recently become commercially available that include specific features to reduce PVL (e.g. Sapien Ultra and Acurate-Neo2) and will need to demonstrate hemodynamic results in future studies.

Peri- and postprocedural complications and 30-day and 12-month mortality rates were mainly similar among THV-groups. Higher rates of major vascular complications after TAVI with Acurate-Neo, Portico and Lotus THV may be due to larger sheath sizes in these procedures or patient-related factors that were not part of this analysis. Rates of permanent pacemaker implantations after TAVI were especially high in patients treated with Lotus THV and, interestingly, patients treated with Sapien-3 and Portico THV had higher rates of new permanent pacemakers compared to patients treated with Evolut and Acurate-Neo THV. This observation contrasts with the findings of previous studies that have shown lower rates of pacemaker implantation after TAVI with balloon-expanding compared with self-expanding THV [19, 20]. Postdilatation was often performed, particularly after implantation of self-expanding THV with a lower radial force. In our analysis, postdilatation was even associated with lower rates of moderate or severe PPM which was in line with previous observations, suggesting optimization of annular geometry [5]. As postdilatation is not associated with increased complication rates (e.g. annular rupture, valve embolization, central aortic regurgitation or pacemaker implantations) [21], it may be a valuable addition in patients at risk of PPM.

This was a retrospective, multicenter analysis, and results can only be interpreted as hypothesis-generating. Randomized trials comparing different THV in patients with a SAA are on the way and will shed further light on optimal treatment options in these patients. Routine clinical variables and imaging data (echocardiography, MDCT) were assessed and reported by the local investigators in default of a central core laboratory. In addition, the accuracy of the depth measurements is strongly dependent on the quality of the final angiography with sufficient contrast filling of the aortic root. Despite a large number of patients, the prevalence of severe PPM was low so that we evaluated moderate and severe PPM together in order to identify independent predictors for their occurrence. A multivariable model corrected for known confounders and repeatedly identified THV design as strong predictor of PPM. Nevertheless, confounding variables may have been missed. Follow-up duration was not sufficient to document a potential implication of PPM on long-term mortality, and longer observation is warranted in these patients.

## Conclusion

In this large contemporary TAVI population, a substantial number of patients presented with a SAA. The prevalence of moderate and severe PPM was high, especially in patients treated with BE and ME THV. SE supra- and intra-annular prostheses demonstrated superior hemodynamics and consequently lower rates of PPM but to increased residual PVL. Severe aortic valve calcification, a SAA, low SVI and a lack of postdilatation were furthermore associated with the occurrence of PPM. In patients with severe aortic stenosis and a small annulus, particularly careful evaluation and device selection may yield superior results after TAVI.

## Supplementary Information

Below is the link to the electronic supplementary material.

Supplementary file1 (DOCX 24 KB)
