# Supplementary material for: Transcatheter aortic valve implantation in patients with a small aortic annulus: performance of supra-, intra- and infra-annular transcatheter heart valves
Source: Clin Res Cardiol. 2021 Aug 13;110(12):1957–66. doi: 10.1007/s00392-021-01918-8 (PMC8639544; doi:10.1007/s00392-021-01918-8)
Supplement: Supplementary file 1 — Supplementary file1 (DOCX 24 KB) [file 392_2021_1918_MOESM1_ESM.docx]

**Table S1: Baseline characteristics according to PPM**

|  | **All**  **(n=867)** | **No PPM**  **(n=533)** | **Moderate PPM**  **(n=243)** | **Severe PPM**  **(n=91)** | **p-value** |
| --- | --- | --- | --- | --- | --- |
| **Clinical data** | | | | | |
| **Age (years)** | 82.7  (79.5, 85.9) | 82.9  (79.9, 86.1) | 82.4  (78.9, 85.7) | 81.3  (78.6, 85.4) | 0.11 |
| **Female gender (%)** | 806 (93) | 494 (92.7) | 225 (92.6) | 87 (95.6) | 0.58 |
| **BSA (m^2^)** | 1.7  (1.6, 1.8) | 1.7  (1.6, 1.8) | 1.7  (1.6, 1.8) | 1.8  (1.7, 1.9) | <0.001 |
| **CAD (%)** | 464 (53.7) | 284 (53.5) | 130 (53.7) | 50 (54.9) | 0.97 |
| **Previous cardiac surgery (%)** | 75 (8.7) | 42 (7.9) | 26 (10.7) | 7 (7.7) | 0.41 |
| **COPD (%)** | 151 (17.4) | 99 (18.6) | 40 (16.5) | 12 (13.2) | 0.41 |
| **Diabetes (%)** | 213 (24.6) | 131 (24.6) | 59 (24.3) | 23 (25.3) | 0.98 |
| **Prior stroke (%)** | 109 (12.6) | 62 (11.6) | 33 (13.6) | 14 (15.4) | 0.52 |
| **Prior malignancy (%)** | 124 (16.1) | 84 (17.8) | 31 (14.7) | 9 (10.7) | 0.22 |
| **STS PROM (%)** | 4.5 (3.1, 6.4) | 4.6 (3.1, 6.6) | 4.4 (3.1, 6.0) | 4.5 (2.8, 6.3) | 0.44 |
| **Echocardiographic data** | | | | | |
| **LVEF <30% (%)** | 575 (66.6) | 352 (66.3) | 167 (68.7) | 56 (62.2) | 0.52 |
| **Stroke volume index (ml/m^2^)** | 49.9  (38.3, 66.0) | 51.0  (40.0, 66.0) | 49.4  (36.8, 71.0) | 44.0  (33.2, 60.7) | 0.02 |
| **iEOA (cm^2^/m^2^)** | 0.4  (0.3, 0.5) | 0.4  (0.3, 0.5) | 0.4  (0.3, 0.4) | 0.3  (0.3, 0.4) | <0.001 |
| **Mean gradient (mmHg)** | 43.0  (32.9, 54.0) | 41.0  (32.0, 51.8) | 45.0  (34.0, 58.0) | 46.5  (33.4, 60.6) | <0.001 |
| **CT Annulus Area (mm^2^)** | 368.0  (342.0, 382.7) | 368.0  (343.0, 382.7) | 368.0  (345.6, 383.0) | 364.8  (333.2, 380.9) | 0.43 |
| **CT Annulus Perimeter (mm)** | 69.3  (67.0, 70.9) | 69.4  (67.0, 70.9) | 69.2  (67.4, 71.1) | 69.2  (66.5, 71.0) | 0.68 |
| **AVC calcification (mm^3^)** | 402.0  (231.6, 633.3) | 367.9  (219.2, 611.3) | 442.0  (245.7, 692.9) | 453.7  (276.5, 698.4) | 0.05 |
| **LVOT calcification (mm^3^)** | 3.5 (0, 32.2) | 3.5 (0, 34.3) | 3.6 (0, 25.2) | 3.4 (0, 41.4) | 0.42 |

Abbreviations: AVC aortic valve complex, BSA body sureface area, CAD coronary artery disease, COPD chronic obstructive lung disease, CT computed tomography, iEOA indexed effective orifice area, LVEF left ventricular ejection fraction, LVOT left ventricular outflow tract, PPM Prosthesis-patient mismatch

**Table S2: Procedural data according to PPM**

|  | **All**  **(n=867)** | **No PPM**  **(n=533)** | **Moderate PPM**  **(n=243)** | **Severe PPM**  **(n=91)** | **p-value** |
| --- | --- | --- | --- | --- | --- |
| **Median THV size (mm)** | 23.0  (23.0, 25.0) | 23.0  (23.0, 25.0) | 23.0  (23.0, 23.0) | 23.0  (23.0, 23.0) | <0.001 |
| **Sizing (according to IFU)**  Undersizing (%)  Within normal range (%)  Oversizing (%) | 21 (2.4)  616 (71.1)  229 (26.4) | 9 (1.7)  379 (71.2)  144 (27.1) | 7 (2.9)  178 (73.3)  58 (23.9) | 5 (5.5)  59 (64.8)  27 (29.7) | 0.08  0.32  0.49 |
| **Relative Oversizing (%)** | 20.4  (9.9, 32.7) | 22.9  (11.1, 32.9) | 15.6  (9.2, 29.4) | 18.7  (8.6, 30.8) | 0.003 |
| **Local anesthesia/**  **conscious sedation (%)** | 679 (79.2) | 420 (79.5) | 185 (77.7) | 74 (81.3) | 0.74 |
| **Procedure time (min)** | 50.0  (34.0, 72.0) | 51.0  (34.0, 72.8) | 44.0  (32.0, 70.0) | 57.5  (37.4, 88.6) | 0.04 |
| **Contrast media (ml)** | 110.0  (75.0, 161.6) | 115.5  (80.0, 164.1) | 100.0  (70.0, 151.0) | 126.0  (76.3, 179.3) | 0.03 |
| **Predilatation (%)** | 467 (54.2) | 291 (55.0) | 125 (51.7) | 51 (56.0) | 0.64 |
| **Postdilatation (%)** | 223 (25.9) | 155 (29.3) | 51 (21.1) | 17 (18.7) | 0.01 |

Abbreviations: IFU instructions for use

**Table S3: Clinical outcomes according to PPM**

|  | **All**  **(n=867)** | **No PPM**  **(n=533)** | **Moderate PPM**  **(n=243)** | **Severe PPM**  **(n=91)** | **p-value** |
| --- | --- | --- | --- | --- | --- |
| **Echocardiographic outcome** | | | | | |
| **iEOA (cm²/m²)** | 0.9 (0.7, 1.1) | 1.0 (0.9, 1.2) | 0.7 (0.7, 0.8) | 0.6 (0.5, 0.6) | <0.001 |
| **Mean gradient (mmHg)** | 10.0 (7.0, 13.0) | 9.0 (6.0, 12.0) | 12.0 (9.0, 16.0) | 13.0 (10.4, 17.6) | <0.001 |
| **PVL > mild (%)** | 43 (5.0) | 32 (6.0) | 10 (4.1) | 1 (1.1) | 0.11 |
| **VARC-2 clinical outcome** | | | | | |
| **Major vascular complications (%)** | 57 (6.6) | 36 (6.8) | 16 (6.6) | 5 (5.5) | 0.90 |
| **Acute kidney injury (%)*** | 66 (8.0) | 35 (6.8) | 19 (8.5) | 12 (13.8) | 0.08 |
| **Major Bleeding (%)** | 50 (5.8) | 32 (6.0) | 12 (5.0) | 6 (6.6) | 0.79 |
| **Permanent pacemaker implantation (%)** | 100 (12.6) | 63 (12.9) | 23 (10.2) | 14 (17.7) | 0.22 |
| **Disabling Stroke (%)** | 6 (0.7) | 4 (0.8) | 2 (0.8) | 0 (0) | 0.70 |
| **Reintervention (%)^+^** | 3 (0.4) | 1 (0.2) | 2 (0.9) | 0 (0) | 0.32 |
| **30 day mortality (%)** | 10 (1.2) | 5 (1.0) | 5 (2.1) | 0 (0) | 0.23 |
| **12 month mortality (%)** | 63 (7.4) | 38 (7.3) | 15 (6.3) | 10 (11.4) | 0.29 |

Abbreviations: iEOA indexed effective orifice area, PVL paravalvular leckage, PPM prosthesis-patient mismatch

* Acute kidney injury `Kidney Disease – Improving Global Outcomes` stadium I-III.

+ Reasons for reintervention: Relevant aortic regurgitation after postdilatation

**Table S4: Association of implantation depth, PPM and PVL**

|  |  | **Implantation depth** | | | **p-value** |
| --- | --- | --- | --- | --- | --- |
|  | **All**  **(n=714)** | **1st tertile (n=252)** | **2nd tertile (n=233)** | **3rd tertile (n=229)** |  |
| **PVL > mild No. (%)** | 33 (4.6) | 12 (4.8) | 9 (3.9) | 12 (5.2) | 0.77 |
| **iEOA (cm²/m²)** | 0.9 (0.7, 1.1) | 0.9 (0.8, 1.1) | 0.9 (0.7, 1.1) | 0.9 (0.7, 1.1) | 0.48 |
| **No PPM (%)** | 444 (62.2) | 160 (63.5) | 139 (59.7) | 145 (63.3) | 0.62 |
| **Moderate PPM (%)** | 195 (27.3) | 67 (26.6) | 66 (28.3) | 62 (27.1) | 0.91 |
| **Severe PPM (%)** | 75 (10.5) | 25 (9.9) | 28 (12.0) | 22 (9.6) | 0.65 |

Abbreviations: iEOA indexed effective orifice area, PVL Paravalvular leckage, PPM Prosthesis-patient mismatch
